# Macronutrient balancing in free‐ranging populations of moose

**DOI:** 10.1002/ece3.7909

**Published:** 2021-07-15

**Authors:** Annika M. Felton, Hilde K. Wam, Adam Felton, Stephen J. Simpson, Caroline Stolter, Per‐Ola Hedwall, Jonas Malmsten, Torsten Eriksson, Mulualem Tigabo, David Raubenheimer

**Affiliations:** ^1^ Southern Swedish Forest Research Centre Swedish University of Agricultural Sciences Alnarp Sweden; ^2^ Division of Forestry and Forest Resources NIBIO Ås Norway; ^3^ Charles Perkins Centre, and School of Life and Environmental Sciences University of Sydney Camperdown NSW Australia; ^4^ Department of Animal Ecology and Conservation Institute of Zoology University of Hamburg Hamburg Germany; ^5^ Department of Wildlife, Fish and Environmental Studies Swedish University of Agricultural Sciences (SLU) Umeå Sweden; ^6^ Department of Animal Nutrition and Management Swedish University of Agricultural Sciences Uppsala Sweden

**Keywords:** *Alces alces*, deer, herbivory, nutritional ecology, primate, ungulate

## Abstract

At northern latitudes, large spatial and temporal variation in the nutritional composition of available foods poses challenges to wild herbivores trying to satisfy their nutrient requirements. Studies conducted in mostly captive settings have shown that animals from a variety of taxonomic groups deal with this challenge by adjusting the amounts and proportions of available food combinations to achieve a target nutrient balance. In this study, we used proportions‐based nutritional geometry to analyze the nutritional composition of rumen samples collected in winter from 481 moose (*Alces alces*) in southern Sweden and examine whether free‐ranging moose show comparable patterns of nutrient balancing. Our main hypothesis was that wild moose actively regulate their rumen nutrient composition to offset ecologically imposed variation in the nutritional composition of available foods. To test this, we assessed the macronutritional composition (protein, carbohydrates, and lipids) of rumen contents and commonly eaten foods, including supplementary feed, across populations with contrasting winter diets, spanning an area of approximately 10,000 km^2^. Our results suggest that moose balanced the macronutrient composition of their rumen, with the rumen contents having consistently similar proportional relationship between protein and nonstructural carbohydrates, despite differences in available (and eaten) foods. Furthermore, we found that rumen macronutrient balance was tightly related to ingested levels of dietary fiber (cellulose and hemicellulose), such that the greater the fiber content, the less protein was present in the rumen compared with nonstructural carbohydrates. Our results also suggest that moose benefit from access to a greater variety of trees, shrubs, herbs, and grasses, which provides them with a larger nutritional space to maneuver within. Our findings provide novel theoretical insights into a model species for ungulate nutritional ecology, while also generating data of direct relevance to wildlife and forest management, such as silvicultural or supplementary feeding practices.

## Graphical abstract

This right‐angled mixture triangle depicts the relative components of macronutrient content in individual moose (*Alces alces*) rumen samples. Our results suggest that the 481 moose in our study balanced the macronutrient composition of their rumen, with the rumen contents having consistently similar proportional relationship between protein and nonstructural carbohydrates, despite differences in available foods. Furthermore, we found that macronutrient balancing was dependent of rumen fiber content, for this large ruminant herbivore.

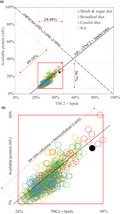

## 1 INTRODUCTION

Wild herbivores living at northern latitudes must deal with large spatial and temporal variation in the nutritional composition of available foods. Despite this variation, individuals must nevertheless obtain sufficient quantities and ratios of nutrients, as demanded and constrained by their own physiology (Felton et al., 2018). None of these nutrients are available as discrete units which the herbivore can freely choose from, but instead come packaged as food items with varying combinations of nutrients and secondary metabolites (including antinutrients). Furthermore, once these items are ingested, the various compounds interact with each other in complicated ways (Villalba & Provenza, 2005).

Due to this complexity, it is becoming increasingly clear that foraging should be seen as a dynamic process which involves balancing the intake of many different nutrients and antinutrients to satisfy complex nutritional needs that change over multiple time scales (Simpson & Raubenheimer, 2012). In recent years, controlled experiments with captive animals have revealed that organisms belonging to diverse taxonomic groups deal with this complexity by selecting foods to achieve a particular target nutrient balance over a given time period (e.g., Dussutour et al., 2010; Hewson‐Hughes et al., 2013; Raubenheimer & Jones, 2006; Raubenheimer et al., 2005; Simpson et al., 2004). They achieve this target balance by continuously regulating the amounts they eat of a variety of foods containing nutrients in specific proportions.

The important question arises as to how the mechanisms of food selection interact with spatial and temporal variation in nutrient availability and accessibility for free‐ranging animals in the wild. Related studies of free‐ranging animals have primarily focused on nonhuman primates, revealing how nutritional balancing governs their food choice (Cui et al., 2018; Felton et al., 2009; Guo et al., 2018; Rothman et al., 2011). Primates, however, are unrepresentative of many species of large herbivore in important respects. First, the primates evolved in, and almost all species still inhabit, the tropics where the foodscape and nutrient availability are relatively stable across seasons (van Schaik & Brockman, 2005). Temperate herbivores, such as cervids (Ungulata, Cervidae), by contrast, experience marked seasonal variation in food and nutrient availability, as well as in energetic requirements for thermoregulation, reproduction, and lactation (Parker et al., 2009). Second, primates are monogastric, and so have a very different physiological relationship with ingested resources than do ruminants such as cervids. Specifically, the foraging choices of ruminants are influenced by the peculiarities of a digestive system characterized by pregastric retention and fermentation with symbiotic microorganisms (Van Soest, 1994).

Cervids are typically large‐bodied and long‐lived, and diet quality and quantity have repeatedly been shown to influence their fitness (Cook et al., 2013; McArt et al., 2009; Proffitt et al., 2016; Saether & Heim, 1993; Wam et al., 2016). Dietary impact on fitness can take place through changes to an individual's body mass (White, 1983), or via maternal nutrition (Langvatn et al., 1996; Saether & Heim, 1993), and produce flow‐on effects spanning several generations (Solberg et al., 2004). Seasonality also plays a significant role in the fitness of cervids, as their body mass and reproduction are strongly influenced by foraging conditions at certain times of the year (Cook et al., 2004; Herfindal et al., 2006; Hjeljord & Histol, 1999; Mysterud & Ostbye, 2006; Proffitt et al., 2016; Stewart et al., 2005; Wam et al., 2016).

**FIGURE 1:**
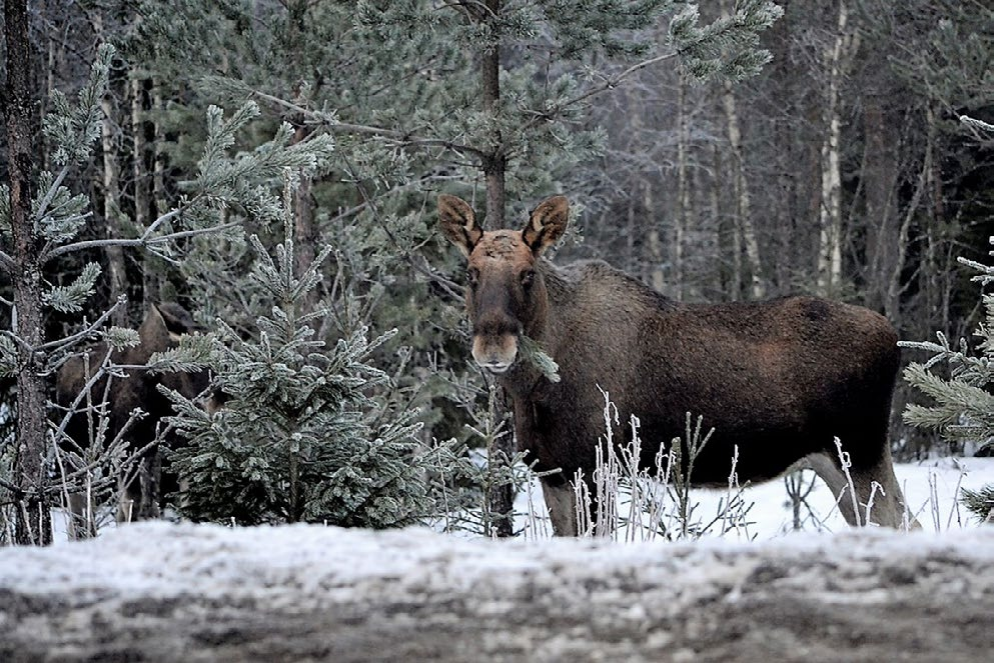
Female moose (*Alces alces*) among broadleaved and conifer food trees. Photo credit: Inger Bjørndal Foss [Correction added on 23 July 2021, after first online publication: Figure 1 caption has been corrected in this version.]

In this paper, we explore nutritional balancing by free‐ranging moose (*Alces alces* L. Figure 1). The moose is a cervid, which lives in the temperate and boreal forests of the northern hemisphere. Because the moose is a ruminant browser, it has a distinct gut morphology adjusted to the summer consumption of leaves and forbs that are relatively easy to digest, and is flexible enough to switch during winter to a diet of twigs, needles and bark (Cederlund et al., 1980; Wam & Hjeljord, 2010) that have a lower nutrient content. A wintertime experiment with captive moose has shown that their food selection is driven by nutrient balancing (Felton et al., 2016), rather than energy or protein maximization as previously assumed (sensu Belovsky (1978) and Mattson (1980)). The moose’ target balance between protein and carbohydrates overlapped with the nutritional composition of the twigs of broadleaved trees commonly eaten by wild moose in wintertime (*Salix* spp.). Results from a recent field study in China using fecal analysis suggest that the balance between nitrogen and nonstructural carbohydrates in winter diets of free‐ranging moose is related to population density (Ma et al., 2020). There are thus indications that foraging in moose, like primates, is driven by nutrient balancing. However, given the important role of fermentation in the rumen of cervids, and the significant variation in the fiber content of forage (Krizsan et al., 2018), we anticipate that if free‐ranging moose regulate their dietary macronutrient balance, then dietary fiber content will play a role. For example, ingested fibers not only provide ruminants with energy through fermentation, as long as nitrogen is available in the feed, but also indirectly provide protein from fiber‐utilizing microorganisms (Dahl et al., 2020). In this study, we used measures of winter rumen content to examine whether free‐ranging moose balance dietary macronutrient contents within the rumen, and determined whether and how this macronutrient balance relates to fiber content.

To obtain a large enough sample to compare diets across a range of foraging environments, we analyzed the contents of winter collected rumen samples of 481 free‐ranging moose belonging to multiple populations in southern Sweden. These populations live in a region with extensive human modification of the landscape (Lindbladh et al., 2014), and the populations face quite different foodscapes primarily defined by intensity of forestry, agriculture, and urbanization, leading to natural browse being highly concentrated in space and time. In addition, many landowners and game managers offer supplementary feed to the game during winter. This feed is typically quite different to woody browse, with higher contents of nonstructural carbohydrates (sugar and starch) (Felton et al., 2017). This further exacerbates differences in the foodscapes of different populations. Diet composition is expected to vary between populations, and the moose within a population may or may not be able to meet their nutritional targets.

To assess the nutritional state of moose living in these varying foodscapes, we used proportions‐based nutritional geometry, an analytical framework for examining nutrient balancing that is particularly well established in the literature of primate nutrition (Raubenheimer, 2011). In this framework, nutrient balancing is indicated if different members of a group (e.g., a species, population, sex, age‐class) compose diets with similar nutritional composition despite eating different combinations of foods (Raubenheimer et al., 2015). Our first hypothesis was that, as in captivity, wild moose regulate the selection and ingestion of food to prioritize a particular balance of nutrients in the rumen content. Multi‐dimensional niche theory, a mixture‐based approach for examining the nutritional niches of animals, predicts that certain dimensions of the diet will be regulated more strongly than others depending on the evolutionary and ecological circumstances of animals (Machovsky‐Capuska et al., 2016). All studies of nutrient balancing in primates to date have demonstrated that the ratio of protein to nonprotein energy (principally nonstructural carbohydrate and lipids) is prioritized by these monogastric herbivores. Our second hypothesis was that the pattern of regulation in the polygastric wild moose would involve not just protein, nonstructural carbohydrate, and lipid, but also the fermentable structural carbohydrates, cellulose, and hemicellulose (Felton et al., 2018; Van Soest, 1994).

## 2 METHODS

### 2.1 Study areas and collection dates

The study area covers about 10,000 km^2^ and is situated between latitude 56° to 59° in the hemiboreal climate zone with mean annual precipitation of 700 mm (see map in Appendix: Figure S1). Forests are dominated by Norway spruce (*Picea abies* L. Karst) or Scots pine (*Pinus sylvestris* L.), sometimes intermixed with naturally regenerated broadleaves: primarily birch, (*Betula pubescens* Ehrh, *Betula pendula* Roth), rowan (*Sorbus aucuparia* L.), aspen (*Populus tremula* L.), and oak (*Quercus robur* L.). Humans largely control the cervid's food resources and also mortality rates as large natural predators are generally scarce or absent. The study region is divided into moose management areas (MMA, Sw: *älgförvaltningsområde*) which are considered to support spatially distinct moose population due to barriers such as fenced highways and major water bodies (Sandström, 2011). Accordingly, we use the term “population” for a MMA. Each MMA is divided into multiple moose management units (MMUs, Sw: *älgskötselområde*), within which the annual hunt takes place. We refer to moose belonging to a MMU as a “subpopulation.” We collected samples from seven MMAs (Appendix: Figure S1) by engaging volunteer hunters active in each of the MMUs.

In this study, we used the same sampled moose individuals as in Felton, Holmström, et al. (2020). Whereas Felton, Holmström, et al. (2020) focused on the botanical composition of moose winter diets, we here focus on the nutritional composition of their rumen content. Sampled moose individuals, of both sexes and all ages, were culled as part of the ordinary hunt, which started on 13 October 2014. However, as our focus was the moose winter diet, dominated by woody material from dormant plants, we restricted some of our data analyses (specified below) to samples collected from 23 October and onwards. By this date, most deciduous trees had lost their leaves, or the leaves largely lacked chlorophyll, and trial checks of rumen contents lacked green leaves. Samples were collected throughout the hunting season (last sample from 22 February). Sampling date distribution was similar across MMAs, with the ratio of samples obtained in early (October–November) versus late (December–February) winter being approximately 70:30. Area A was an exception with proportions 30:70 (Felton, Holmström, et al., 2020). We also included samples from traffic‐injured moose within the study areas (7 individuals in total). All individuals were sampled in the same way, regardless of sex or age.

Data were collected regarding location, sex, and carcass weight (dressed carcass, after removal of skin, head, blood, metapodials, and internal organs; hereafter “body mass”, BM). Hunters also collected the lower jaw from each moose that was not a calf or yearling. To estimate the moose’ age, we sectioned one first‐molar tooth and counted the cementum layer (Wolfe, 1969). We defined calves as individuals born the previous spring (6–9 months old at sampling); yearlings as individuals born one year earlier; and adults as all individuals born ≥2 years before sampling. Among the sampled moose, 52% were calves, 14% yearlings, 20% adult females, and 14% adult males (Felton, Holmström, et al., 2020).

We used calf body mass as an index of subpopulation body mass status (for information about body mass of other age classes, see (Felton, Holmström, et al., 2020)). In Scandinavia, calf body mass is generally linked to cow body mass (smaller cows produce smaller calves, and smaller calves become smaller adults) and population productivity such as calf production per cow (Pettorelli et al., 2005; Solberg et al., 2004; Tiilikainen et al., 2012). Indeed, among our sampled subpopulations, mean calf body mass was positively related to the number of calves observed per adult female (Felton, Holmström, et al., 2020). Furthermore, calf body mass may be the most representative sample of the population body mass as it circumvents age effects on BM (Ericsson et al., 2001; Sand, 1996) and calves are less affected by hunter bias in the sex and size of individuals harvested (Moe et al., 2009). Mean calf BM per subpopulation was calculated based on data collected from 23 October 2014 to 22 February 2015 (Table S1). There was no correlation between calf body mass and harvesting date (Pearson correlation, *N* = 203 calves, *r* = −.043).

### 2.2 Background information on the sampled moose’ winter diet types

To aid our interpretation of results regarding rumen nutritional composition, we refer to our previously published results on differences among the same study animals in the botanical composition of rumen contents (Felton, Holmström, et al., 2020). To analyze the botanical composition, frozen rumen samples were defrosted, mixed, and analyzed through macroscopic analysis (as per Nichols et al., 2016) to identify plant fragments to the lowest taxonomic level possible (hereafter “plant categories”). As described by Felton, Holmström, et al. (2020), the winter diet was on average characterized by relatively large proportions (% dry matter (dm)) of needles and wood from *Pinus sylvestris* (30% of dm), twigs from three dwarf shrubs *Vaccinium vitis‐idaea* L. (9%)*, Calluna vulgaris* L. (9%), and *V. myrtillus* L. (7%), and the three broadleaved tree species/genera *Salix* spp (8%), *Quercus robur* (7%), and *Betula* spp (6%). In total, 63% of dm was material from trees and bushes, 28% from dwarf shrubs (i.e., *Vaccinium* spp and *C*. *vulgaris*) and 9% from forbs, grasses and root vegetables. Three distinct diet types among study subpopulations were identified, as well as differences in the diversity of plant categories in the diet (Table 1). In Felton, Holmström, et al. (2020), it was also found that the three diet types were associated with variation in subpopulation mean calf body mass. The diet type we refer to as the “conifer diet” was associated with relatively low body mass; the “shrubs and sugar diet” with intermediate body mass; and the “broadleaf diet” with higher body mass (Table S1).

**TABLE 1:** A general description of three distinct winter diet types identified among the moose in this study

	Description of diet	Mean species richness *(per sample)*	MMA (# MMU)
Broadleaf Diet	A diverse mix of plants including relatively large shares of broadleaved trees. *Pinus sylvestris* (24% dm) and *Salix* spp (13% dm) provided the largest shares, while *Salix* spp* and *Populus* spp* most strongly characterized the diet statistically	28 *(7.9)*	B (5), D (4), E (4) and *F* (3)
Shrub and Sugar Diet	A mix between sugar‐rich crops and forest items. *P. sylvestris* (23% dm) and *Vaccinium vitis‐idaea* (17% dm) provided the largest shares, while root vegetables*, Calluna vulgaris*, *V. vitis‐idaea, V. myrtillus*, and narrow‐leaved grass most strongly characterized the diet statistically	29 *(8.6)*	G (3), D (4), E (1)
Conifer Diet	A relatively species poor diet dominated by conifer species. *P. sylvestris* (44% dm) and *C. vulgaris* (9%) provided the largest shares, while Juniperus communis** and P. sylvestris** most strongly characterized the diet statistically. Unusually high proportions *P. abies* (4%) and grass silage (5%)	23 *(6.5)*	A (3) and C (3)

Note

**FIGURE 2:**
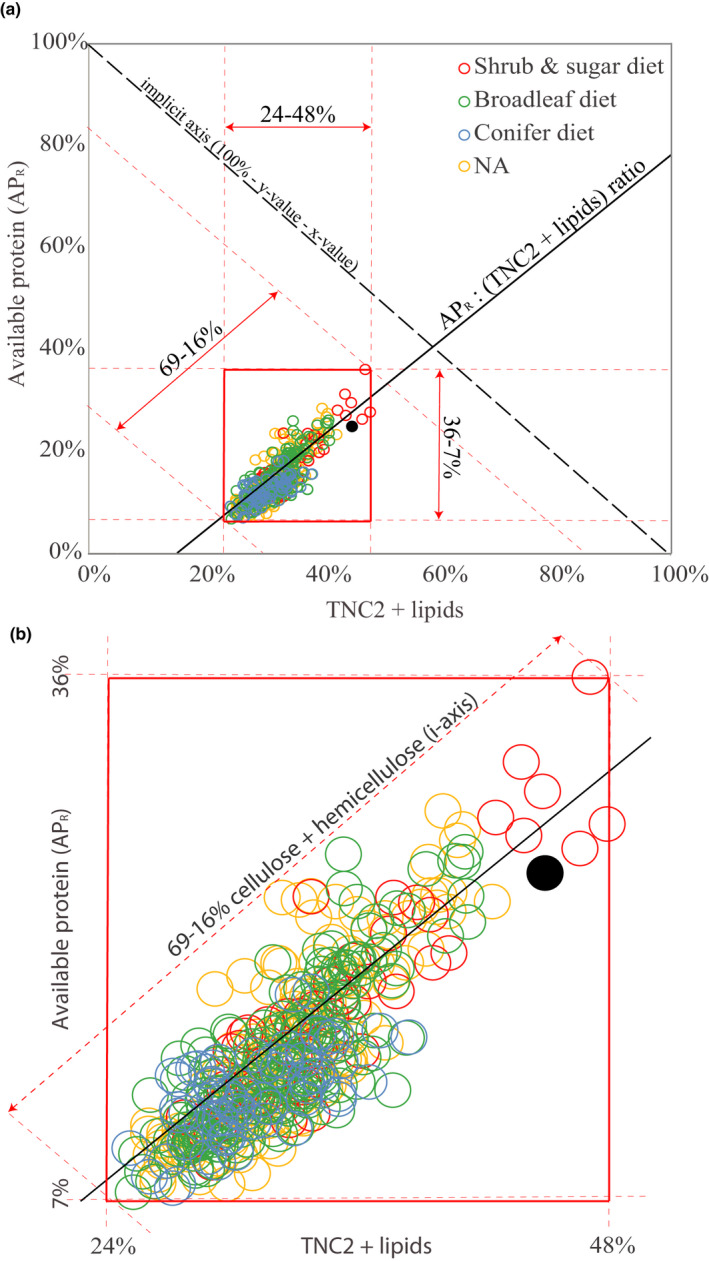
(a) Right‐angled mixture triangle (RMT) (Raubenheimer, 2011) depicting the relative components of macronutrient content in individual moose rumen samples (*n* = 481) collected during the winter 2014/15 in southern Sweden, expressed as a percentage of total macronutrients in g dry matter (dm). X‐axis = % macronutrients providing highly digestible energy (a combined measure of total nonstructural carbohydrates and lipids (TNC2+lipids)). Y‐axis = % available protein (AP_R_) which includes microbial‐N. I‐axis (implicit axis, i.e., 100% minus y‐value minus x‐value) = % cellulose and hemicellulose (fibers). Increased distance from the hypotenuse means increased % fiber. Circles = composition of individual rumen samples. For example, the filled black circle represents a sample (approx. one meal) with contributions of 25% AP, 30% fiber, and 45% TNC2+lipids, totaling 100%. Solid black line: linear relationship between (nonstandardized) % AP_R_ and %TNC2+lipids (y = 0.98x − 0.17, *R*² = 0.70). Dashed red lines draw the upper and lower limits of the three dimensions’ observed ranges, while solid red lines mark the resulting range of observations, which is expanded in panel (b). (b) Three diet types (distinguished by color) were identified in these moose populations through rumen analysis, as classified by a previous study (Table 1 and Felton, Holmström, et al., 2020)). NA (yellow circles) indicates rumen samples where such plant identification data are not available. Note that no representatives of the “conifer diet” had less than 45% fiber (of total macronutrients), while the other two diet types were represented across the entire range of % fiber

**FIGURE 3:**
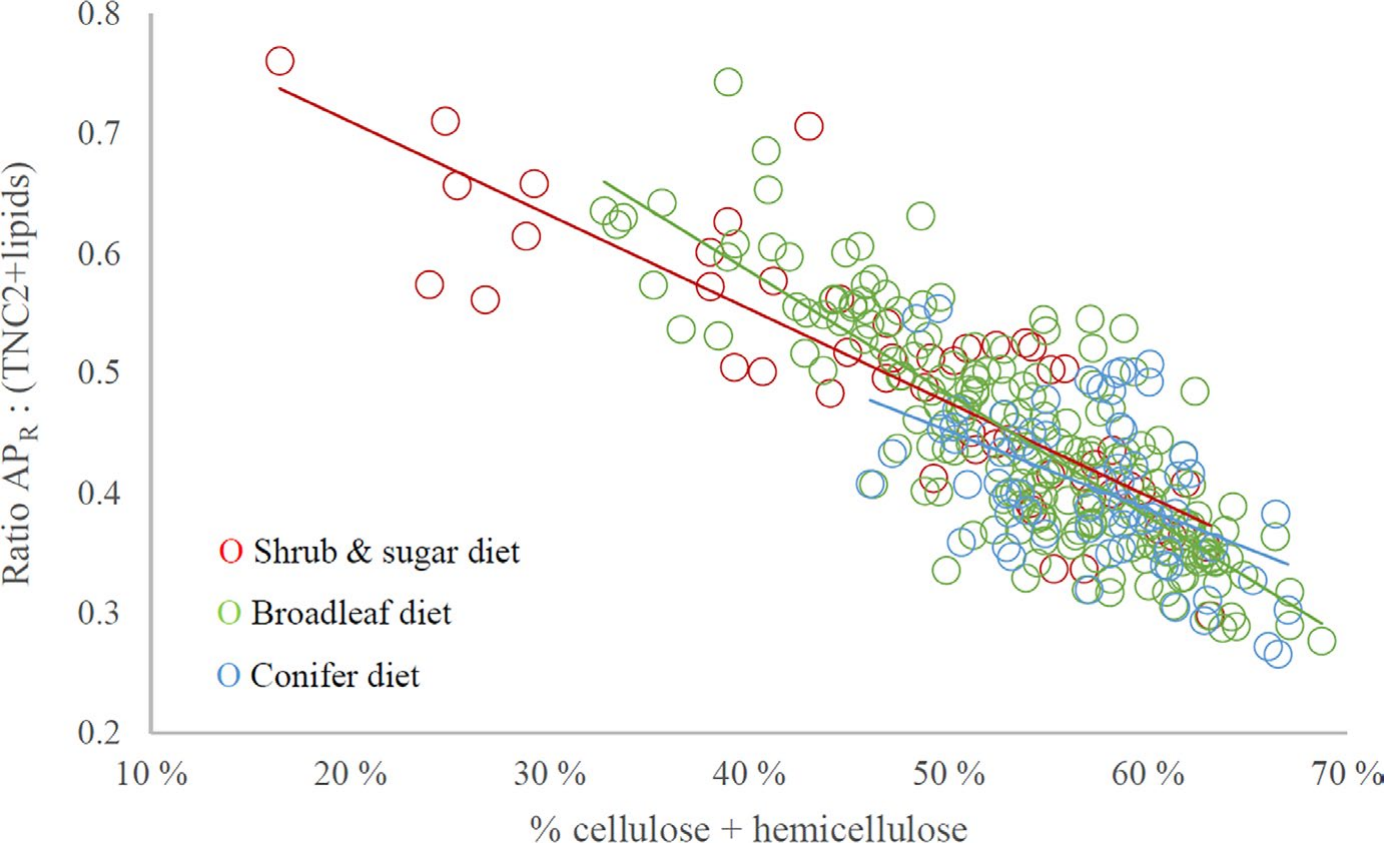
Relationship between the proportion of fiber (cellulose and hemicellulose) and the ratio between available protein (AP_R_) and macronutrients providing highly digestible energy (TNC2+lipids (see Figure 2)), in individual moose rumen samples (*n* = 319) collected during the winter 2014/15 in southern Sweden. Values are based on percentages of total macronutrients in g dry matter (dm), where AP_R_, TNC2+lipids, and fiber sum up to 100%. Lines are drawn for the linear relationship of each of the three diet types, where the “broadleaf” diet type had a steeper slope than the “shrub and sugar diet” (t = 3.1, *p* = .002, see Table S3 for parameter coefficients). Note how the range of fiber contents differed between the diet types

Plant categories were identified through macroscopy of rumen contents (described in Felton, Holmström, et al., 2020). The list of species provided here is not exhaustive but includes species that characterize each diet type, by proportion and statistical relevance. The diet types were associated with certain moose management areas (MMA; populations A‐G (Appendix, Figure S1)) and their moose management units (MMU, subpopulations, the number of MMU per MMA with relevant diet indicated in bracket). Mean species richness is defined as the total number of plant categories identified in the respective diet, across all moose subpopulations representing the diet type, and across the period 23 October 2014 to 31 January 2015. Also displayed (*italics* in brackets) is the mean number of plant species per rumen sample. Colors correspond to Figure 2 and Figure 3.

*these variables are most strongly positively associated with subpopulation mean calf body mass (in a PCA including all subpopulations for which there was at least 5 rumen samples with macrohistological results), while **these plant categories are most strongly negatively associated with subpopulation mean calf body mass (Felton, Holmström, et al., 2020).

### 2.3 Rumen samples

#### 2.3.1 Moose sample collection for rumen contents

To obtain information about the nutritional composition of foods eaten, fresh rumen samples were collected immediately after harvest by filling a 1‐L plastic airtight container with rumen content. Hunters were instructed to mix contents and take material from throughout the rumen (Cederlund et al., 1980) and to remove excess rumen liquid by squeezing each handful. Rumen samples were frozen shortly after sampling (normally 0.5–1 hr) and stored at −20°C. Rumen samples were dried at 60°C until the samples came to a constant mass and then ground using a hammer mill (KAMAS^©^ Slagy 200B; 1 mm sieve).

We obtained 481 rumen samples (collected between 13 October 2014 and 22 February 2015, Appendix: Table S2), all of which were used in our sample‐based nutritional analyses (wet chemistry, near infrared spectroscopy (NIRS), and right‐angle mixture triangles). For other data analyses, we restricted the data to those MMUs for which we had at least 5 individual rumen samples collected from the 23 October and onwards (30 subpopulations, *N* = 301 samples in total, Appendix: Table S1).

#### 2.3.2 Chemical analyses of rumen content

Due to high costs associated with wet chemistry analyses, we used NIRS to estimate the concentrations of nutritional constituents of rumen samples, with a subset of representative samples also analyzed using wet chemistry for calibration purposes (as per Vance et al., 2016). Each ground rumen sample was thoroughly mixed before drawing ca. 40 g for scanning. NIRS reflectance spectra were acquired with XDS Rapid Content Analyzer (FOSS NIRSystems, Inc.) from 780 to 2,498 nm at an interval of 0.5 nm. After scanning a total of 481 samples, the most representative samples were selected for chemical analyses based on scores of principal component analysis (PCA) (see Tigabu & Felton, 2018). In brief, samples were selected as representative (i.e., spanning the entire range of variation in concentrations of nutritional constituents) depending on the distance from the center of the data in all three principal components. This resulted in 148 samples selected as representatives with regard to all nutritional parameters except microbial‐N, for which 111 samples were selected (Tigabu & Felton, 2018).

Selected samples were analyzed for dm, ash, Kjeldahl nitrogen (total N), crude fat (hereafter called “lipids”), neutral‐detergent fiber (NDF), acid‐detergent fiber (ADF), and lignin using conventional wet chemistry techniques (see Appendix S1 Supplementary methods) described by Bertilsson et al. (2017). To estimate the portion of nondigestible protein, we measured insoluble nitrogen remaining in acid‐detergent fiber (ADF‐N). Available protein (AP) was then calculated as total protein (total N multiplied by 6.25, due to the average N content of proteins being ca 16% (1/0.16 = 6.25)) minus nondigestible protein (ADF‐N multiplied by 6.25) (Licitra et al., 1996). Hemicellulose was calculated as NDF–ADF and cellulose as ADF–lignin.

Because the length of time that foods were in the rumen was unknown, as was their extent of digestion before sampling, any estimate of quickly digestible constituents, such as total nonstructural carbohydrates (TNC) and lipids, must be treated with caution. Therefore, instead of devoting resources to chemically analyzing these for the rumen content, we estimated them by subtraction: TNC + lipids = 100 − (NDF + AP + ash) (Irwin et al., 2014) and hereafter refer to this estimation as TNC2. For the same reasons, rumen samples were not analyzed for in vitro organic matter digestibility.

Of the nitrogen in rumen samples, some will be undegraded food protein and some will be microbial‐N. The proportions between them change during the digestion of a meal. While both fractions contribute to the ruminant's protein supply, it is important to note that their relative proportions when absorbed postruminally are not the same as in the rumen sample (Van Soest, 1994). We denote total N minus ADF‐N as “available nitrogen” (Avail N). “Available protein” was calculated as Avail N multiplied by 6.25 and abbreviated as AP_R_ to acknowledge that the indigestible part of the microbes’ nitrogen content (typically ca 20%, Storm et al., 1983) is not identified by ADF‐N analysis, contrary to indigestible feed protein (Van Soest, 1994). To estimate microbial nitrogen (microbial‐N, the total nitrogen in rumen content that originated from microbes), we determined total purine content as per Zinn and Owens (1986), with modifications according to Aharoni and Tagari (1991), with yeast‐RNA (Roche 10,109,223,001) as a standard. This yielded a result in mg yeast‐RNA equivalents/g sample that was subsequently converted to microbial‐N by multiplying with the factor 1.1 (as per Volden & Harstad, 1998; more details in Appendix S1 Supplementary methods).

Results from the wet chemistry analyses of the representative subset of rumen samples (*n* = 148) were used to establish multivariate calibrations of NIRS spectra (see Tigabu & Felton, 2018). After handling outliers in the dataset (see Appendix S1, Supplementary methods), we divided the dataset into a calibration set for training the model (*n* = 118 for crude and available protein, *n* = 117 for ash, *n* = 113 for NDF, and *n* = 81 and 74 for microbial‐N and lignin, respectively) and a prediction set to validate the fitted models (*n* = 30 for crude and available protein, ash, NDF and ADF, and *n* = 21 for microbial‐N and lignin). The calibration models were derived by Orthogonal Projection to Latent Structure (OPLS) and applied to the remaining rumen samples (*n* = 333), with a prediction error of 0.4, 0.7, and 0.5% dm, respectively, for crude protein, available protein AP_R_ and ash (*R* ^2^ = 0.99, 0.96 and 0.92). The 333 samples in question had been excluded during calibration calculations. The other constituents were predicted with slightly higher prediction error but acceptable accuracy (see Tigabu & Felton, 2018): NDF (*R* ^2^ = .92; prediction error = 2.2% dm), ADF (*R* ^2^ = .89; 1.9% dm), lignin (*R* ^2^ = .84; 1.1% dm), and the microbial‐N marker purine (*R* ^2^ = .81; prediction error = 1.3 mg yeast‐RNA g–1 dm). In the following data analyses, we used predicted values for all nutritional variables of all samples, with a few exceptions (Appendix S1, Supplementary methods), for which we used wet chemistry results.

### 2.4 Plant samples

#### 2.4.1 Sample collection of plants

To frame our interpretation of the rumen data, we assessed the nutritional composition of plants commonly eaten by moose in the study region during winter (Cederlund et al., 1980; Spitzer, 2019). We collected twigs from trees and shrubs growing in 11 unfertilized young production stands dominated by *Pinus sylvestris* (tree height 0.5–3 m), located in two of the MMAs included in the study (D and E). The collection was done in mid‐March 2015, and included 12 plant species: *P*. *sylvestris, P. abies, B. pendula, B. pubescens, S. aucuparia, P. tremula, Salix caprea* L.*, Q. robur, Juniperus communis* L.*, V. vitis‐idaea, V. myrtillus*, and *C. vulgaris*. These 12 plant species together represent ca 85% of the total dry matter ingested by these populations (Felton, Holmström, et al., 2020). The plants were dormant at the time of collection, and we assumed that their nutritional composition at this time was roughly representative for the majority of the winter period, acknowledging that some plant metabolic activity occurs during dormancy, especially during mild winters (Perry, 1971). For tree species, one indiscriminately selected nonbrowsed side shoot per individual was cut from branches 0.5–2 m above ground (for sample size see Table 2), of a diameter usually consumed by moose from the plant species in question (p. 7 in Felton et al., 2016; p. 1,298 in Persson et al., 2005). For dwarf‐shrub species (*V. vitis‐idaea, V. myrtillus, C. vulgaris*), we cut handfuls of twigs of approximately 3–5 cm in length from the top layer of the plants to mimic the feeding behavior of moose. We pooled all collected material from each plant species across stands and MMAs, thereby incorporating between‐tree and between‐site variation in nutritional content. The 12 samples were weighed before drying at 60°C until the samples came to a constant mass, and then ground using a hammer mill (KAMAS^©^ Slagy 200B; 1 mm sieve).

**TABLE 2:** The winter nutritional composition of 12 moose food plants, in terms of the percentage of total dm of crude protein (CP), available protein (AP), total nonstructural carbohydrates (TNC1), lipids, lignin (indigestible fiber), cellulose and hemicellulose, neutral‐detergent fiber (NDF), and the digestible fraction of NDF (dNDF)

Species	*n*	diam	CP	AP	TNC1	lipids	Lignin	Cellul	Hemi	NDF	dNDF
*Betula pendula*	10	2.0	8.8	5.7	5.7	4.0	18.1	29.9	12.9	57.7	19.5
*Betula pubescens*	10	2.1	8.3	5.9	5.3	1.0	16.7	31.4	15.0	60.2	19.3
*Calluna vulgaris*	1	na	7.8	6.1	10.6	2.2	12.5	23.3	8.3	42.1	24.3
*Juniperus communis*	3	1.7	6.8	5.4	8.5	3.1	10.8	30.2	3.6	42.6	19.7
*Picea abies*	11	2.5	8.2	7.4	15.8	2.0	9.8	21.2	10.6	40.1	20.8
*Pinus sylvestris*	11	2.3	9.2	8.4	10.1	4.1	9.5	25.6	9.7	43.1	21.3
*Populus tremula*	6	2.3	7.4	5.5	5.9	1.5	13.5	32.4	13.2	57.1	26.4
*Quercus robur*	9	2.1	6.6	4.3	7.3	0.2	18.3	30.5	13.5	59.2	21.3
*Salix caprea*	8	2.4	7.7	5.7	6.2	0.7	18.5	31.6	8.6	56.6	24.9
*Sorbus aucuparia*	11	2.7	6.5	5.1	5.6	0.9	13.9	27.7	12.8	52.2	18.3
*Vaccinium myrtillus*	4	na	6.1	3.6	11.3	1.5	20.6	24.9	6.6	49.0	20.2
*Vaccinium vitis‐idaea*	5	na	6.5	3.3	13.9	2.5	19.8	21.9	4.5	44.7	21.5

Note

Together these 12 food plants represented 85% of the mean intake (in terms of dm in rumen content) of moose included in this study (Felton, Holmström, et al., 2020). We sampled plant material (twigs, with needles for conifers) in young forests (0.5–3 m tree height) in southern Sweden, March 2015. *n* = number of individual plants sampled per species which equals the number of stands (of the 11 stands visited) in which the species occurred. The individual plants were pooled per species before nutritional analysis (thus no standard deviation). diam = mean diameter of the twigs collected (mm; not applicable (na) for *Vaccinium myrtillus, V. vitis‐idaea*, and *Calluna vulgaris*).

#### 2.4.2 Chemical analyses of plants

Plant samples were analyzed for ash, total nitrogen, NDF, ADF, and lignin using the same wet chemistry methods described above for rumen samples. We also conducted the same analysis of ADF‐N to calculate available protein (AP) in the plant samples. Cellulose and hemicellulose were calculated in the same way as described for rumen samples. For plant samples, we estimated total nonstructural carbohydrates (TNC) by determining water soluble carbohydrates (sugars) and starch enzymatically (Larsson & Bengtsson, 1983), and summed those two values. We refer to this estimate as TNC1 to clarify the methodological difference of estimation of TNC of rumen samples, for which we used the subtraction method (TNC2, see above). We also analyzed in vitro organic matter digestibility of the plant material, using conventional wet chemistry techniques (see Appendix S1, Supplementary methods) described by Bertilsson et al. (2017). We report estimates of the digestible fraction of plants’ NDF (dNDF), based on this in vitro analysis.

In addition, we used published data on the nutritional composition of five common supplementary foods previously identified in the rumen content of these moose individuals (Felton, Holmström, et al., 2020), namely whole roots of *Beta vulgaris, Solanum tuberosum* and *Daucus carota,* the peas of *Pisum sativum*, and haycrop (grass) silage with <25% legume content. For *B. vulgaris*, data on available protein and TNC were found in Spörndly, (2003) and data on NDF in Eriksson et al. (2009). For the remaining four items, we used data in Spörndly, (2003). Note that the data for grass silage in Spörndly, (2003) were based on pooled data from a large number of Swedish farms and likely over‐represents silage aimed for dairy cow management, and therefore is likely of higher digestibility and energy content than supplementary silage fed to game.

It is important to note that we did not aim to perform statistical or in‐depth assessments of the relationships between plant compositions and rumen macronutrient composition, because the chemical composition of the rumen contents may differ significantly due to transformations taking place in the rumen (Van Soest, 1994). For example, fiber fractions will be retained in the rumen until they have been sufficiently fermented and degraded to pass out. In contrast, easily digestible carbohydrates can either be directly absorbed by the animal or immediately fermented to volatile fatty acids and leave through the rumen wall relatively quickly, as will nitrogen fractions if degraded to ammonia. Likewise, if there is a nitrogen shortage in the food intake, urea recycling will buffer nitrogen levels in the rumen. We therefore present the nutritional data of rumen contents and plants separately and use the plant composition data only to frame our discussion of the rumen data.

### 2.5 Statistical analyses

Statistical analyses were done in Minitab (Minitab 17 Statistical Software, 2010) unless specified otherwise. When interpreting results of nutritional patterns, it is important to know how the concentrations of nutritional constituents covary with each other (Felton et al., 2018; Van Soest, 1994). We used a correlation matrix (Pearson correlation) to analyze the potential covariation among all nutritional constituents in rumen contents. The same was done for the 12 food plants.

Differences among MMUs in sex and age ratio among sampled moose may have been influenced by locally set hunting quotas. To assess whether the age or sex of the individual moose had any bearing on our results, we tested whether there were differences in the nutritional composition of rumen content or assigned diet types between the age‐sex classes (*n* = 350), using one‐way ANOVAs separately for each of the following response variables: ash, available protein, cellulose, hemicellulose, microbial‐N marker, lignin, TNC2+ lipids, and the three diet types: the “broadleaf diet”, the “conifer diet” or the “shrub and sugar diet” (Table 1). ANOVA was also used to test differences in microbal‐N and lignin in rumen content (both mg/g, and as % of total g macronutrient) between subpopulations (*n* = 30; Appendix: Table S1) identified as having either diet type.

To illustrate how the food plants that are generally available to moose in their foraging landscape differed from each other nutritionally, we performed principal component analyses (PCA) of nutritional compositions (% dm) of each of the 12 plant species we collected in field. Constituents included were lignin, cellulose, hemicellulose, available protein, total nonstructural carbohydrates, in vitro digestibility of NDF, and lipids. We conducted a similar PCA for five common supplementary foods and the 12 plant species in combination, but with only three of the nutritional constituents (due to a lack of published data): available protein, total NDF, and TNC.

To test our first hypothesis, we visualized the macronutrient and fiber content of rumen samples using right‐angle mixture triangles (RMT). The RMT included all rumen samples (*N* = 481)—that is, both moose harvested inside and outside of the plant sampling areas—to incorporate as much variation as possible. RMTs represent three‐component compositions of nutrient mixtures as two‐dimensional Cartesian points (Raubenheimer, 2011). This allowed us to examine relationships among the dietary components most relevant to moose nutrition (Felton et al., 2018): 1) available protein, 2) cellulose + hemicellulose, and 3) TNC2+ lipids (i.e., the sum of total nonstructural carbohydrates and lipids). All values are expressed as % of total macronutrients (dm sum of the three‐component groups). Because lipids alone contributed little to this total, given its consistently low content (≤4%) in the majority of food items (Table 2), we combined them with the more abundant and variable TNC in the RMTs (as per Johnson et al., 2015). Because of the different range of data for each variable, we standardized data (subtracting the mean and dividing by the standard deviation) before testing whether the relationship between the variables was linear and scaled isometrically (in this case 1:1, because of standardization). We used linear regression to test whether the ratio between available protein and TNC+lipid per subpopulation (*n* = 30) was related to the subpopulation mean calf body mass. To test our second hypothesis, we also used linear regression to see whether the ratio between available protein and TNC+lipid varied with fiber content across the entire dataset. Finally, we also tested whether the linear relationship between the ratio APR: (TNC2+lipids) and % fiber interacted with the three diet types (“lm” in the R software version 3.5.1; nonstandardized data) (RCoreTeam, 2018). In addition, we ran this test using standardized data and generalized linear models (quasi‐binomial distribution, due to the bounded proportional data). Both solutions gave similar results, and we present the linear models as these can be more directly interpreted.

## 3 RESULTS

### 3.1 The nutritional composition of rumen contents

Moose of different age‐sex classes did not differ markedly in their rumen nutritional composition. There was no significant difference among the six age‐sex classes (*n* = 181 females (86 calves, 28 yearlings, 67 adults) and 169 males (101 calves, 21 yearlings, 47 adults)) for six of the seven nutritional constituents: ash (ANOVA, *F* = 1.64, *p* = .148), AP_R_ (*F* = 1.58, *p* = .166), lignin (*F* = 1.71, *p* = .131), cellulose (*F* = 2.00, *p* = .078), hemicellulose (*F* = 1.49, *p* = .192), and microbial‐N marker (*F* = 1.03, *p* = .400; DF = 349 for all constituents). However, there was a significant difference among age‐sex classes in the proportion of TNC2+lipids (*F* = 2.90, *p* = .014), and a Tukey's Pairwise Comparison showed that male and female calves (24.1% and 24.2%, respectively) had significantly higher %TNC2+lipids than adult females (content 23.2%), likely because of calves still had a small intake of milk at the time of sampling. However, due to the overall similarity among age‐sex classes, we grouped data from all age‐sex classes in subsequent analyses. The distribution of the age‐sex classes was similar across the three diet types (Figure S2; there were no significant difference in the proportion of any of the six age‐sex classes among subpopulations representing the three diet types).

The nutritional constituents measured in individual rumen samples were strongly correlated with each other (Table 3). Notably, one of the two strong negative correlations evident in food plants (Table 4) was found as a positive correlation in the rumen samples: available protein and lignin.

**TABLE 3:** Correlation coefficients from Pearson correlation of the covariation among seven nutritional constituents (% of dm) measured in individual moose rumen samples (*n* = 481) collected during the winter 2014/15 in southern Sweden

	Ash	Lignin	microbial‐N	APR	Cellulose	Hemicellulose
Lignin	0.783					
microbial‐N	0.536	0.798				
AP_R_	0.750	0.956	0.853			
Cellulose	−0.886	−0.962	−0.752	−0.938		
Hemicellulose	−0.658	−0.786	−0.669	−0.796	0.780	
TNC2+lipids	0.720	0.696	0.482	0.640	−0.805	−0.802

Note

All correlations were significant (*p* < .0001). The nutritional constituents included are ash, lignin, microbial‐N (estimated from total purine analysis), “available protein” (AP_R_), cellulose, hemicellulose, and the combined measure TNC2+lipids (see Methods).

**TABLE 4:** Results from Pearson correlation of the covariation among six nutritional constituents (% of dm) measured in 12 important moose food plants during winter in southern Sweden

	Lignin	Cellulose	Hemicellulose	AP	TNC1
Cellulose	0.181				
	*0.574*				
Hemicellulose	0.005	0.451			
	*0.988*	*0.141*			
AP	**−0.790**	−0.062	0.256		
	*0.002*	*0.848*	*0.422*		
TNC1	−0.196	**−0.909**	−0.557	0.051	
	*0.542*	*0.000*	*0.060*	*0.876*	
lipids	−0.380	−0.284	−0.341	0.432	0.253
	*0.223*	*0.372*	*0.278*	*0.161*	*0.427*

Note

The nutritional constituents included were available protein (AP), total nonstructural carbohydrates (TNC1), lipids, lignin (indigestible fiber), cellulose, and hemicellulose. For each pairwise comparison, the correlation coefficient (above) and *p*‐value (below, italics) are listed. Significant correlations (*p* < .05) are in bold.

The concentration of microbial‐N marker in the rumen samples ranged between 0.4 and 2.3 g/100g dm and represented on average 49% (range 20%–77%) of the total available *N* (see Appendix S1, Supplementary discussion). The mean microbial‐*N* (% of total dm) per subpopulation was significantly different among the three diet types identified (*N* = 26 subpopulations; *F* = 8.56, *p* = .001). A Tukey pairwise comparison showed that the “shrub and sugar diet” had higher levels of microbial‐*N* (0.92% of total dm) than the “broadleaved” (0.71%) and “conifer” diets (0.70%). The “shrub and sugar diet” also had significantly higher % lignin (18%) than the “broadleaf” (16%) and “conifer” diets (16%) (*N* = 26 subpopulations; *F* = 25.49, *p* < .001).

### 3.2 Nutritional balance of rumen contents

Examination of rumen nutrient composition using right‐angle mixture triangle (RMT, Figure 2) illustrates that the nutritional composition of rumen samples fell within the following range of our three compositional groups (percent of total macronutrients, in g dm): 7%–36% AP_R_, 24%–48% highly digestible macronutrients (TNC2+lipids), and 16%–69% cellulose and hemicellulose combined (henceforth referred to as “fiber” for simplicity; note that lignin is not included). Within this range, there was a tight linear relationship between % AP_R_ and % TNC2+lipids (*R* ^2^ = .70, β = 0.84 ± 0.025, t = 33.3, *p* < .001; standardized data). Although the relationship (AP_R_ = 0.84 (TNC2+lipids)) scaled statistically isometric (in this case 1:1, because of standardized variables), the deviance from a slope of 1 was sufficient to create a statistical decrease in the ratio AP_R_: (TNC2+lipids) as % fiber increased in the mix (Figure 3, R^2^ = .68 β = −0.97 ± 0.031, t = −31.5, *p* < .001). There was a significant interaction between % fiber and diet type (*p* ≤ .001, Table S3). Moose with the “broadleaf diet” experienced a stronger drop in AP_R_: (TNC2+lipids) the more fiber their forage contained, compared with moose on the “shrub and sugar diet” (Table S3).

The “shrub and sugar diet” and the “broadleaf diet” were both represented across the full range of variation in fiber (Figure 2b), while the “conifer diet” was consistently found only in the high fiber range (>45% of all macronutrients). Furthermore, samples with the lowest % fiber (and highest AP_R_:(TNC2+lipid) ratio) were representatives of the “shrub and sugar diet” (Figure 2b). There was no significant relationship between the AP_R_:(TNC2+lipid) ratio and subpopulation mean calf body mass (DF = 28, t = −0.149, *p* = .88).

### 3.3 The nutritional composition of commonly eaten food plants

**FIGURE 4:**
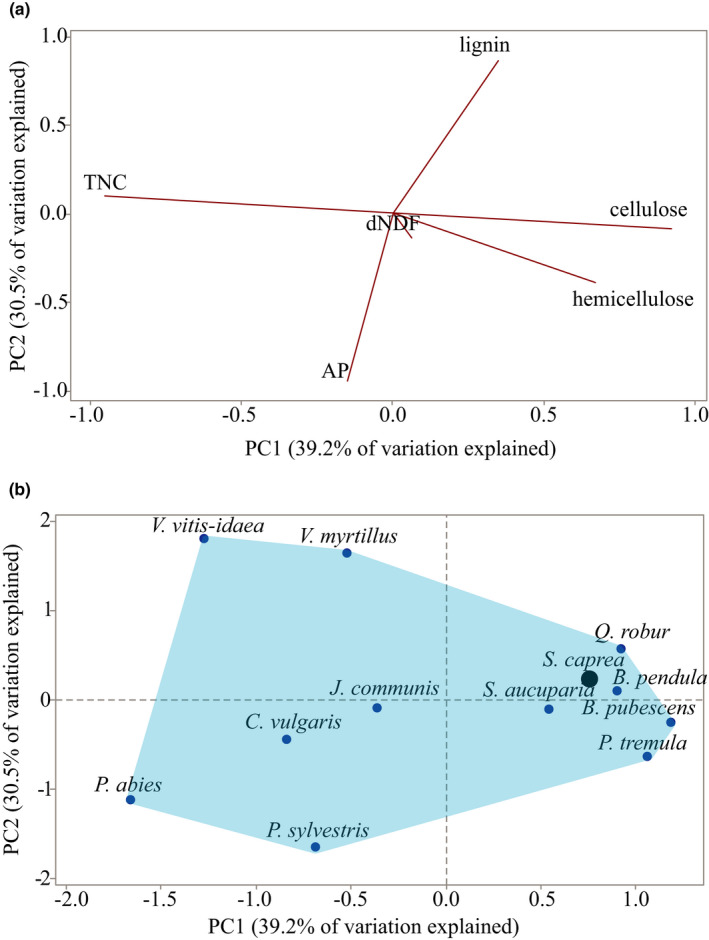
(a) PCA results illustrating the nutritional composition of 12 common moose food plants in Sweden, using the mean composition (% dm, estimated by wet chemistry) of six nutritional constituents (loading plot). Edible parts of twigs and/or needles edible were sampled in winter (March 2015). The first principal component (PC1, x‐axis) depicts variation in total nonstructural carbohydrates (TNC1, increasing values to the left), cellulose, and hemicellulose (both with increasing values to the right). The second principal component (PC2, y‐axis) depicts variation in lignin (increasing values upwards) and available protein (AP, increasing values downwards). In vitro digestibility of NDF (dNDF) has neutral values on both axes. For loading values, see Appendix: Table S4. (b) Score plot from the same PCA showing the placement of the 12 plant species within this nutritional space. Six of the plant species are evergreen: *Pinus sylvestris, Picea abies, Juniperus communis, Vaccinium vitis‐idaea, Calluna vulgaris*, and *V. myrtillus* (even though the latter is deciduous). *Salix caprea* is denoted by a large black point, as its nutritional composition has been found to correspond to the wintertime nutritional target balance of moose, as identified experimentally with captive moose (Felton et al., 2016). A moose that has access to all plants has a larger nutritional space (blue field) to navigate within compared with a moose that only has access to a few of the plants. The 12 food items together represent ca 85% of total ingested dry matter by these moose populations (Felton, Holmström, et al., 2020)

The nutritional composition varied among the 12 commonly eaten food plants sampled in the field (Table 2). There were, however, two pairs of nutritional constituents in the food plants that were consistently correlated: available protein was negatively correlated with lignin, and total nonstructural carbohydrates (TNC1) was negatively correlated with cellulose (Table 4). These correlations are illustrated by the two first PCA components that together explained 70% of the variation in the data (Figure 4a; Appendix: Table S4). Within the score plot of this PCA, plant species showed distinct clustering: twigs from the six broadleaved trees *B. pendula, B. pubescens, S. aucuparia, P. tremula, S. caprea*, and *Q. robur* had similar nutritional compositions (Figure 4b). The dwarf bushes *V. vitis‐idaea* and *V. myrtillus* had a higher proportion of lignin and lower AP than the other plant species. Needles and twigs of *P. sylvestris* and *P. abies* had more AP and less lignin (PC2) than the broadleaved twigs, but also a different ratio between their structural and nonstructural carbohydrates (PC1).

**FIGURE 5:**
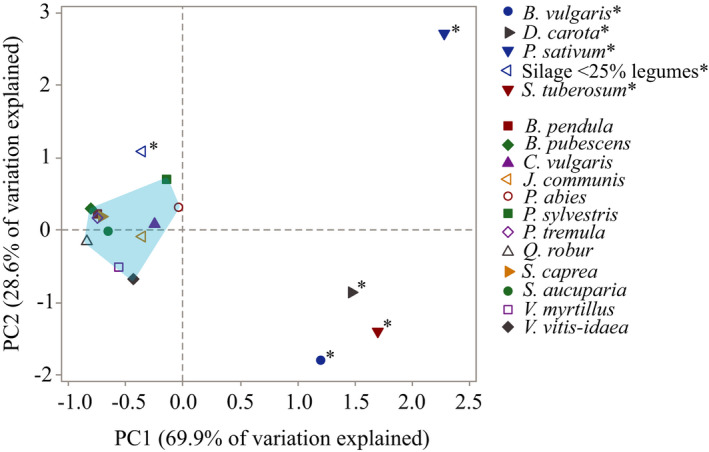
The score plot from a PCA of the nutritional composition of five common supplementary feeds (marked with asterisk) used in Sweden and 12 common moose food plants (parts of their twigs edible for moose). The supplementary feeds are three root vegetables (whole roots of *Beta vulgaris, Solanum tuberosum*, and *Dauca carota*), one type of silage (a grass mix with <25% legumes), and green peas (*Pisum sativum*). Included in the PCA are the nutritional constituents (as % dm): available protein, NDF, and total nonstructural carbohydrates (TNC1). The first principal component (PC1, x‐axis) depicts variation in NDF (increasing values to the left) and TNC1 (increasing values to the right; more details in Appendix: Table S5 and Figure S3). The second principal component (PC2, y‐axis) depicts variation in protein (increasing values upwards). The blue field illustrates the nutritional space created by the 12 moose food plants that are not supplementary foods (see Figure 4)

A comparison between the 12 natural food plants in Figure 4 and five supplementary foods, using three nutritional constituents (available protein, total NDF, and TNC), shows that the two types of food had distinct nutritional compositions (Figure 5). The two first components of the PCA we used to assess this together explained 98% of the variation in the data (loading values in Appendix: Table S5, loading plot in Figure S3). The natural food plants had lower proportions of TNC (5%–16% TNC of dm) compared with the root vegetables (74% starch of dm in *S. tuberosum*; 65% sugar of dm in *B. vulgaris*; 60% sugar of dm in *D. carota;* sugar refers to the sum of sucrose, fructanes, free glucose, and free fructose (Spörndly, 2003)). The location of the grass silage in the PCA shows that it had a carbohydrate composition more consistent with natural food plants, but with higher protein concentrations.

## 4 DISCUSSION

A key finding of this study was that across the rumens of 481 moose there was a consistent relationship between nonstructural carbohydrates and protein, even though the study covered some 10,000 km^2^ of varying foraging conditions. Taken together with evidence for macronutrient balancing by captive moose (Felton et al., 2016), and indications of nutrient balancing from fecal analysis (Ma et al., 2020), our study confirms nutritional balancing by a ruminant herbivore species in the wild. Furthermore, the results confirm that macronutrient balancing is dependent on rumen fiber content.

During the winter, diet choices for moose in the Northern hemisphere are limited, so the moose have to make the best of a more restricted choice of food options compared with summer time (Wam & Hjeljord, 2010). There has been ambiguous empirical data and partly conflicting theories about the nutritional underpinnings of these choices (reviewed by Felton et al., 2018). Our findings here from free‐ranging moose in southern Sweden support earlier conclusions from moose in captive settings that their food selection during wintertime is governed by macronutrient balancing as opposed to alternative strategies such as energy or protein maximization (Felton et al., 2016). Particularly, it appears that their pattern of macronutrient regulation focuses on balancing protein (AP_R_) and highly digestible macronutrients (TNC plus lipids) (Figure 2). Lipids likely played a minor role in this interplay as both plants and rumens were low in lipids. Notably, the proportions of protein and TNC were not correlated in the 12 food plants we assessed as representing the natural food types (i.e., excluding supplementary feeds and crops) generally available to these moose. This suggests that the moose selected combinations of foods in amounts that provided the observed balance between protein and TNC—that is, they demonstrated macronutrient balancing. This finding supports our first hypothesis. Although the moose ate many more plant items than those 12 during the winter, together these 12 food plants represented ca 85% on average of total rumen dry matter for these populations (Felton, Holmström, et al., 2020).

The observed pattern of macronutrient balancing by moose in wild settings, with an emphasis on a balanced intake of protein in relation to TNC, complement studies that have suggested homeostatic regulation of energy intake in moose and closely related deer species in captivity. For example, moose (Schwartz et al., 1988), fallow deer (Weber & Thompson, 1998), and red deer (Asher et al., 2011; Webster et al., 2000) have been found to maintain a stable intake of digestible energy despite significant variation in food composition. While the ruminal balance between protein and TNC was tightly regulated by moose in our study, there was large variation in cellulose and hemicellulose. Wild moose in Norway likewise appear to be more flexible in their intake of hemicellulose than of protein and TNC when selecting among available birch leaves, one of their main staple foods in summer (Wam et al., 2018). To achieve such balancing of protein and TNC, through a regulatory flexibility of structural carbohydrates, moose need access to plants with sufficient variation of all these macronutrients (urea recirculation also contributes partly to regulation of rumen protein (Van Soest, 1994)). Analyses of available food plants in our study indicated that concentrations of TNC and cellulose +hemicellulose were strongly negatively correlated (Table 4). Hence, gaining a target balance between protein and TNC from food plants containing low concentrations of TNC inevitably required the moose to consume more cellulose and hemicellulose, and vice versa. While cellulose and hemicellulose are essential dietary components for ruminants, and can provide them with up to 80% of ingested energy (Barboza et al., 2008), these dietary constituents require long retention (Van Soest, 1994), and handling times for the animal (cropping, chewing, and rumination; Perez‐Barberia & Gordon, 1998; Shipley & Spalinger, 1992). Their influence on the food selection of moose and other northern cervids is therefore repeatedly reported to be negative, albeit there are also several studies showing a positive influence on food selection (reviewed in Felton et al., 2018).

Even though the pattern of nutritional regulation in the wild moose in this study had an emphasis on a balanced intake of protein in relation to TNC, we also found support for our second hypothesis, as cellulose and hemicellulose (“fiber” for simplicity) are nonetheless intricately involved. When we examined the protein to TNC relationship (Figure 3), we found that as the proportion of fiber increased across rumen samples, the protein:TNC ratio significantly decreased (consistent with the relation between protein and TNC having a regression slope <1; Figure 2). This importance of fiber contrasts with what has been observed in primates. For example, in the face of seasonal changes in fruit availability, frugivorous spider monkeys maintain a stable protein intake while allowing their nonprotein energy intake (starch, sugars, and lipids) to vary (Felton et al., 2009), while the opposite pattern is observed in the folivorous mountain gorilla (Rothman et al., 2011). A significant influence of fiber is not demonstrated in either case.

We suggest that the contrasting pattern between these primates and moose is due to the complexities of the ruminant digestive system. Although our data represent only one region and one season, and we thus cannot extrapolate the observed outcomes to all moose, we believe there are general physiological differences that warrant discussion about the potential underlying explanations. In the moose rumen, when % fiber is high, and % protein is relatively low, the fiber‐fermenting rumen microbes may be too N‐limited to convert food substrate into bacterial biomass (Hoover, 1986). Microbial growth may also be inhibited by the lack of readily available carbohydrates, which all rumen microbes require (Van Soest, 1994). On the contrary, too small an intake of structural carbohydrates, or too abrupt changes in them, can affect the rumen microbes negatively (Gordon et al., 2002; Tomkins et al., 1991). Overly rapid fermentation, caused by high concentrations of nonstructural carbohydrates, can result in a decline in pH which reduces microbial efficiency (Sniffen et al., 1992) and fiber digestion (Pitt et al., 1996). Indeed, if a ruminant's carbohydrate intake is shifted too far toward starch and sugars, ruminal acidosis can occur, with negative implications for digestion, milk production, overall condition, and in severe cases, death (Keunen et al., 2002; M.V.M, 2005).

Across our moose rumen samples, low concentrations of cellulose and hemicellulose appear to have been compensated for by intake of higher concentrations of lignin (Table 3). This may explain why, instead of a negative effect on microbial growth, we found that rumen samples with low % cellulose and hemicellulose (and high % TNC) had relatively high % microbial‐N, which in turn, may explain the higher protein to TNC ratio at this end of the fiber range (Figure 3). It is generally agreed that rumen energy supplies are the main driving force for microbial protein production (Broderick et al., 2010; Clark et al., 1992). In fact, moose with the “shrub and sugar diet” (with high % TNC) had higher levels of microbial‐N in their rumen than other moose in our study. This diet type included sugar‐rich root vegetables (commonly used as supplementary feed in this region (SOU, 2014)), which are very different from the natural winter foods of moose (Figure 5). We emphasize, however, that these moose ate rather small proportions of root vegetables (mean 8% of dm (Felton, Holmström, et al., 2020)) and that a lot of shrubs were also included in their diet which must have boosted lignin intake, and therefore likely sustained a relatively stable environment in the rumen (Allen, 1997).

We did not find a relationship between the rumen protein: TNC ratio and subpopulation mean calf body mass. Our results thus appear not consistent with previous results where a higher N:C ratio in the vegetation during winter was associated with higher moose population densities (Ma et al., 2020). However, moose in the subpopulations with the higher calf body mass may still have had a higher *absolute* intake of N than moose in the subpopulations with lower calf body mass. Interestingly, the subpopulations with the lowest calf body mass in our study had a “conifer diet”, and there were higher concentrations of available protein in conifer winter browse than in broadleaf browse (Figure 4). There are three potential drivers of such a seemingly contradictive outcome.

First, food availability may have been limited. Previous research has shown that the relatively low mean calf body mass of the conifer diet subpopulations is correlated with low diversity of plant items, and low availability of young regenerating forest; a habitat type rich in deciduous browse (Felton, Holmström, et al., 2020). Indeed, this estimate of forage availability was a strong explanatory factor of the variation in calf body mass among all subpopulations in that study. Second, if the available food is low in energy and protein, and simultaneously high in plant secondary metabolites, the ruminant's absolute food intake may also be reduced (Villalba & Provenza, 2005). The fact that this diet was dominated by conifer material (higher proportions of *P. sylvestris, P*. *abies*, and *J. communis* than the other two diets (Table 1)) is of particular interest. Coniferous plants are generally only selected for by Scandinavian moose if they do not have high access to deciduous browse (e.g., Wam & Hjeljord, 2010). This could partly be due to some conifers containing different and often higher concentrations of digestion inhibitors (mainly terpenoids but also phenolics) than deciduous browse (which mainly have phenolics, in lower concentrations) (Bryant et al., 1991, 1992; Stolter et al., 2009). We speculate that these moose may have ingested high doses of defensive chemicals that inhibit digestion (as in Radwan, 1972). A third possible reason why the “conifer diet” moose subpopulations had relatively low calf body mass despite a similar balance between AP and TNC in their rumens is that these moose had eaten relatively large proportions of grass silage (Table 1), which is associated with a particular fiber structure (different from fibers in browse) that slows down gut passage in captive moose (Lechner et al., 2010; Renecker & Hudson, 1990) and may lead to weight loss in moose (Schwartz & Hundertmark, 1993). The latter two hypotheses (digestion inhibitors in conifer browse and presence of grass silage) can both help us understand the odd mismatch between the consistently high % fiber of “conifer diet” rumen samples and the relatively low % fiber of conifer browse compared with broadleaved browse (Figure 4). However, any empirical links between the plant and rumen data should be treated with caution, due to transformations taking place in the rumen.

Our results have potential implications for landscape and wildlife management. While efforts to increase the diversity and availability of different food plants should benefit moose in southern Sweden, not all plant items have an equal nutritional value. Our earlier research indicates that there is a disproportionate benefit to moose from the availability of broadleaves, such as the genera *Salix*, *Populus*, *Sorbus*, and *Quercus,* compared with conifers (Felton, Holmström, et al., 2020), and other studies have shown that these broadleaf tree genera are highly selected by moose in Scandinavia during wintertime (Månsson et al., 2007; Shipley et al., 1998; Wam & Hjeljord, 2010). The results from this study suggest that the nutritional composition of the twigs of these broadleaved tree species (Figure 4) likely contributes to this high degree of selection, as it resembles the identified protein:TNC balance targeted by captive moose (Felton et al., 2016).

In contrast, attempts to increase dietary diversity via supplementary feeding requires caution, due to a range of potentially unintended and negative outcomes (Milner et al., 2014; Sorensen et al., 2014, and this study). For example, a significant dose of imbalanced supplementary feed may cause moose to try to compensate with contrasting items such as conifers or broadleaved browse (Figure 5). In an environment where the most readily available complementary plant material is young stems of pine or spruce (e.g., in dense dark production forests where the cover of dwarf shrubs is low (Felton, Löfroth, et al., 2020; Hedwall & Brunet, 2016)), these stems are likely to be used for fiber compensation, with associated damage and financial losses for the forest owner. If supplementary feeding is deemed necessary regardless, hay silage appears to be a more appropriate option from a macronutritional perspective, as the composition of silage is closer to the moose’ natural winter diet than root vegetables and peas (Figure 5). However, even the silage option should be used with caution, if the target species is moose, because grass is not a large part of the natural diet of wild moose (Spitzer et al., 2020). Moose cannot so efficiently digest grass (Lechner et al., 2010), and long‐term feeding on grass has been found to trigger adverse digestive reactions in captive moose (Shochat et al., 1997). Grass silage may be a more suitable supplement for intermediate feeders, such as red deer (*Cervus elaphus*) and fallow deer (*Dama dama*), that normally include larger portions of grass in their diet (Spitzer et al., 2020).

Some caveats are associated with the interpretation of our results. Our data represent one year, season, and geographical region. Interannual variation in food nutritional contents and availability can be large (Wam et al., 2016), and the moose’ nutritional strategy could differ among years and among regions. Furthermore, because the absolute amounts of food eaten by individuals was unknown, as was the extent of digestion at the time of sampling, any estimate of quickly digestible constituents must be treated with caution. As we used proportions‐based nutritional geometry, an error in the estimation of one parameter affects the relative value of another parameter. In addition, although our estimates of microbial‐N resemble those from comparable studies of dairy cows (Bertilsson et al., 2017; Bertilsson & Murphy, 2003; Eriksson et al., 2004), there are some uncertainties related to how well our method estimated the amount of microbial‐N/mg purine marker in moose rumen. Much of what is known about interactions between diet and rumination comes from studies of domestic grazers, whose digestive physiology differs significantly from the moose (Clauss et al., 2011).

We recommend future research to investigate the relationship between diet and microbial processes in the rumen of wild browsers, and to explore the relative roles of diet selection versus physiological homeostasis processes (Van Soest, 1994) in explaining the nutrient balance we can observe in solid rumen material. Further research is also needed on how micronutrients and plant secondary metabolites (PSM) add dimensions to the nutritional space available to them (see Wam et al., 2018). It is possible that some unidentified parameter (e.g., mineral or PSM) covaried with the rumen constituents assessed, masking the true drivers. We suggest, however, that it is more likely that such unidentified factors would cause more noise in our data (larger cloud in Figure 2), rather than less. Further research is needed to clarify why the relationship between the protein:TNC ratio and % fiber (in Figure 3) was significantly different depending on the diet type. Furthermore, although the 12 forage species analyzed represented ca 85% of the dry matter ingested on average, they did not represent the complete diets, especially not the more species‐rich diets. It would be of value for researchers to build up a library of comparable nutritional data from more forage species for future comparisons. Finally, how the nutritional balance of diet, diversity of food plants, and forage quantity interact and vary in their relative influence on moose *fitness* remains an open question and more research is required to tease out further details of this complexity, for this species and other members of the family Cervidae.

Answers to these questions will significantly enrich the comparative study of nutrient balancing by free‐ranging herbivores and enhance the understanding of the complex relationship between macronutrients and fiber observed in our study. Above all, identified relationships between food environments, diet choice, and animal fitness provide practical and powerful information for landscape and wildlife management.

## CONFLICT OF INTEREST

The authors declare no competing interests.

## AUTHOR CONTRIBUTIONS

**Annika M. Felton:** Conceptualization (lead); Data curation (lead); Formal analysis (lead); Funding acquisition (lead); Investigation (lead); Methodology (equal); Project administration (lead); Resources (lead); Validation (equal); Visualization (equal); Writing‐original draft (lead); Writing‐review & editing (lead). **Hilde K. Wam:** Conceptualization (supporting); Formal analysis (supporting); Validation (supporting); Writing‐original draft (supporting); Writing‐review & editing (supporting). **Adam Felton:** Conceptualization (supporting); Investigation (supporting); Methodology (supporting); Validation (supporting); Writing‐original draft (supporting); Writing‐review & editing (supporting). **Stephen J. Simpson:** Formal analysis (supporting); Investigation (supporting); Methodology (supporting); Validation (supporting); Writing‐review & editing (supporting). **Caroline Stolter:** Conceptualization (supporting); Investigation (supporting); Validation (supporting); Writing‐original draft (supporting); Writing‐review & editing (supporting). **Per‐Ola Hedwall:** Formal analysis (supporting); Investigation (supporting); Validation (supporting); Visualization (supporting); Writing‐review & editing (supporting). **Jonas Malmsten:** Conceptualization (supporting); Data curation (supporting); Funding acquisition (supporting); Investigation (supporting); Methodology (supporting); Writing‐review & editing (supporting). **Torsten Eriksson:** Investigation (supporting); Methodology (supporting); Validation (supporting); Writing‐review & editing (supporting). **Mulualem Tigabo:** Formal analysis (supporting); Methodology (supporting); Software (supporting); Validation (supporting); Writing‐review & editing (supporting). **David Raubenheimer:** Conceptualization (supporting); Formal analysis (supporting); Investigation (supporting); Methodology (supporting); Visualization (equal); Writing‐original draft (supporting); Writing‐review & editing (supporting).

## ADDITIONAL INFORMATION

The methods used in this study comply with the current laws of Sweden.

## Supporting information

Supplementary Material

## Data Availability

Raw data on the macronutritional composition of moose rumen samples are available at https://doi.org/10.5061/dryad.9zw3r22fh.
