# Supplementary material for: Macronutrient balancing in free‐ranging populations of moose
Source: Ecol Evol. 2021 Jul 15;11(16):11223–40. doi: 10.1002/ece3.7909 (PMC8366896; doi:10.1002/ece3.7909)
Supplement: Supplementary file 1 — Supplementary Material [file ECE3-11-11223-s001.docx]

# Appendix

**Supplementary Methods**

Chemical analyses of rumen content

To estimate microbial nitrogen we determined total purine content as per Zinn & Owens (1986), with modifications according to Aharoni and Tagari (1991), with yeast RNA (Roche 10109223001) as a standard. This yielded a result in mg yeast RNA equivalents/g sample that was subsequently converted to microbial N by multiplying with the factor 1.1, established in previous analyses of isolated cattle rumen bacteria at the same laboratory with the same methods (Volden and Harstad, 1998). When interpreting results it is important to note that the rumen pool is not identical to the outflow from the rumen and the subsequent uptake of amino acids in the small intestine (Clark et al., 1992). To what extent microbial protein contributes to the actual protein supply in the moose we cannot tell with our data, as it depends on outflow rates of different fractions and on how well our conversion factor estimates microbial N. We used a static value for the conversion, but there is a possibility for dietary differences in the true relationship between microbial N and microbial purines.

Chemical analyses of plants

We analysed concentrations of dm, ash, Kjeldahl nitrogen, crude fat (lipids), neutral-detergent fiber (NDF), acid-detergent fiber (ADF), lignin, and 96-hour in vitro organic matter digestibility in rumen liquid from dairy cows, using conventional wet chemistry techniques described by Bertilsson et al. (2017). Residual moisture in all ground material was determined by oven drying at 103 °C for 16 h. Ash (total minerals) was determined by ignition at 550 °C for 3 h. Nitrogen concentration was determined by the Kjeldahl method using a 2020 Digestor and a 2400 Kjeltec Analyser Unit (FOSS Analytical A/S, Hillerød, Denmark). ADF and lignin were determined by using the detergent system of Van Soest for fiber analysis (Van Soest et al., 1991). Samples were analyzed for ash-free NDF using heat stable α-amylase and sodium sulphite, and crucibles instead of filter bags (Chai and Uden, 1998).

Data analysis – handling outliers in the NIRS data set

Preliminary calibration revealed 1%, 2%, 3% and 7% of the samples for microbial-N, ash, NDF and ADF, respectively had high Hotelling’s T2 (a statistic used in multivariate quality control charts), residuals that were more than twice the pooled standard residuals (a statistic used to determine lack of fit of the model), or observed values that differed by more than 3 standard deviations from the predicted values. Thus, these samples were outliers and removed from the final calibration. In our data analyses, we used predicted values for all constituents of all samples, with the following exceptions: for five samples concerning lignin, and for 24 samples concerning microbial N we used observed wet chemistry values instead, due to the criteria of outlier identification described above.

**Supplementary Discussion**

Rumen microbial N

The microbial N part was on average 49% of available N in the rumen pool in this study, which is similar to the rumen pools of dairy cows with microbial N constituting 39 – 70% of total N if the same conversion factor is applied (Bertilsson and Murphy, 2003, Bertilsson et al., 2017). The range of microbial N concentrations we found among our rumen samples (4-23 mg/g) could be compared to treatment means of 12 – 19 mg/g DM for lactating dairy cow ruminal contents analysed with the same methods (Eriksson et al., 2004, Bertilsson and Murphy, 2003, Bertilsson et al., 2017).

**Table S1.** Mean proportion of eight nutritional constituents as percent of dry matter in rumen content per moose subpopulation (MMU, n = 30). Each MMU belongs to one of seven populations (MMA A-G). Included in the table are MMUs for which rumen samples from at least 5 individuals were obtained (n = 301 samples in total), collected between 23-Oct-2014 and 22-Feb-2015. For each MMU the mean calf carcass body mass (Calf), the number of rumen samples (n), and diet type (Diet) are listed. The nutritional constituents include ash, crude protein (CP), available protein (AP_R_; total N minus ADF-N), microbial nitrogen (estimated from total purine analysis), total non-structural carbohydrates and lipids (TNC+lipids, by subtraction), cellulose, hemicellulose and lignin.

| MMA | MMU | n | Diet* | Calf (kg) | ash | CP | AP_R_ | microbialN | TNC+lipids | cellulose | hemicellulose | lignin |
| --- | --- | --- | --- | --- | --- | --- | --- | --- | --- | --- | --- | --- |
| A | 35 | 6 | C | 51,3 | 5,8 | 11,6 | 9,9 | 7,4 | 23 | 30,6 | 14,5 | 16,1 |
| A | 37 | 10 | C | 53,5 | 6,5 | 11,5 | 9,4 | 6,5 | 21,8 | 31,4 | 15,3 | 15,6 |
| A | 40 | 10 | C | 54,3 | 6,9 | 12,9 | 11,2 | 8,3 | 24,2 | 27,2 | 13,5 | 17,1 |
| B | 50 | 9 | B | 59,7 | 7 | 12,8 | 10,7 | 8,6 | 22,2 | 28,7 | 15 | 16,3 |
| B | 51 | 11 | B | 54,1 | 8,3 | 13,7 | 11,5 | 7,8 | 24,5 | 24,2 | 14,1 | 17,3 |
| B | 52 | 7 | B | 59,5 | 6,6 | 12,6 | 10,6 | 8,8 | 23,2 | 28,8 | 14,6 | 16,1 |
| B | 53 | 5 | B | 62,4 | 6,3 | 12 | 10,1 | 7,3 | 23,7 | 29,2 | 14,6 | 16,1 |
| B | 56 | 5 | B | 64,8 | 7,4 | 14 | 11,8 | 8,4 | 24,6 | 25 | 13,8 | 17,4 |
| C | 25 | 17 | C | 52,9 | 6,7 | 11 | 9,1 | 7 | 24,3 | 30,3 | 14,4 | 15,2 |
| C | 42 | 8 | C | 52,9 | 7,1 | 11,4 | 9,4 | 6,8 | 24,6 | 28,6 | 14,4 | 15,9 |
| C | 44 | 18 | C | 56,4 | 6,4 | 10,8 | 9 | 6,2 | 22,8 | 31,7 | 14,6 | 15,4 |
| D | 1 | 11 | B | 62,8 | 6,7 | 12 | 10,1 | 7,6 | 24,1 | 28,7 | 14 | 16,3 |
| D | 2 | 5 | B | 57,9 | 8,1 | 12,5 | 10,2 | 6,8 | 24,1 | 26,6 | 14,6 | 16,5 |
| D | 3 | 7 | S | 59,5 | 6,9 | 11,7 | 9,7 | 7,1 | 23,7 | 28,7 | 14,9 | 16,1 |
| D | 4 | 7 | S | 54,9 | 6,1 | 10,5 | 8,5 | 5,9 | 24,4 | 31,3 | 14,6 | 15,1 |
| D | 5 | 8 | S | 61,4 | 6,3 | 10,9 | 8,8 | 5,8 | 22,6 | 31,6 | 15,5 | 15,2 |
| D | 6 | 10 | B | 63,7 | 7,3 | 12,7 | 10,6 | 7,6 | 24,9 | 26,3 | 14,1 | 16,7 |
| D | 7 | 21 | B | 63,7 | 6,8 | 11,1 | 8,9 | 6,4 | 23,5 | 30,7 | 14,9 | 15,2 |
| D | 62 | 10 | S | 58,5 | 7,2 | 11,8 | 9,9 | 6,6 | 24,1 | 27,7 | 14,8 | 16,3 |
| Cont. Table S1 | | | | | | | | | | | | |
| MMA | **MMU** | **n** | **Diet*** | **Calf (kg)** | **ash** | **CP** | **AP_R_** | **microbialN** | **TNC+lipids** | **cellulose** | **hemicellulose** | **lignin** |
| E | 11 | 5 | B | 61 | 7 | 11,7 | 9,6 | 6,9 | 23,2 | 30,2 | 14,1 | 16 |
| E | 12 | 6 | B | 62 | 7,1 | 12,3 | 10,5 | 7,3 | 23,4 | 28,2 | 14,4 | 16,4 |
| E | 13 | 8 | B | 60,2 | 6,6 | 10,5 | 8,4 | 5,4 | 24,3 | 31,4 | 14,5 | 14,9 |
| E | 15 | 11 | S | 62,8 | 6,8 | 11 | 9 | 6,8 | 23,9 | 30,4 | 14,5 | 15,4 |
| E | 16 | 11 | B | 60,2 | 8 | 13 | 10,9 | 7,8 | 24,3 | 25,4 | 14,4 | 17 |
| F | 21 | 9 | B | 59,1 | 5,5 | 10,4 | 8,6 | 7 | 21,6 | 34,3 | 15,3 | 14,7 |
| F | 22 | 15 | B | 59,5 | 7,2 | 12,4 | 10,5 | 6,9 | 24,5 | 27,3 | 14 | 16,6 |
| F | 23 | 6 | B | 68,8 | 7 | 12,3 | 10,4 | 6,8 | 23,4 | 28,3 | 14,4 | 16,4 |
| G | 31 | 8 | S | 56,2 | 7,9 | 13,9 | 12,1 | 8,8 | 24,8 | 24 | 13,5 | 17,6 |
| G | 33 | 26 | S | 57,2 | 8,6 | 14,9 | 13 | 9,4 | 25,2 | 21,3 | 13,5 | 18,3 |
| G | 34 | 11 | S | 53 | 8,5 | 15 | 13,3 | 9,4 | 26,3 | 20,1 | 13,1 | 18,7 |

*Diet type B = Broadleaf diet; C = Conifer diet; S = Shrub & sugar diet; see Table 1 Main text for description.

**Table S2.** Samples from moose in southern Sweden were collected in seven moose management areas (MMA, each representing a moose population), divided into multiple moose management units (MMU; subpopulation). The number of MMU from which we obtained samples is indicated (^a^), as well as their summed area (^b^) in terms of tha (1000 ha), mean area (^c^) and the proportion of the MMA which was included in the study (^d^). Samples were collected from moose harvested between 13-Oct-2014 and 22-Feb-2015. The full data set (sample size B, n = 481) was used in our sample-based analyses of nutritional composition and nutritional balancing (wet chemistry/ NIRS and right-angle mixture triangles). Based on a more limited data set (sample size A, n = 350, which excludes individuals harvested before 23-Oct-2014 and samples lacking data regarding either sex, body mass or age (^e^)), we tested potential differences in rumen composition among age-sex classes of moose.

| Moose management area (MMA) | A | B | C | D | E | F | G | Total |
| --- | --- | --- | --- | --- | --- | --- | --- | --- |
| Area (tha) | 73 | 487 | 87 | 141 | 130 | 87 | 169 | 1176 |
| Number of MMU^a^ | 6 | 18 | 4 | 8 | 9 | 6 | 9 | 60 |
| Sum MMU area (tha)^b^ | 61 | 245 | 70 | 141 | 108 | 76 | 91 | 793 |
| Mean area of MMU (tha)^c^ | 10 | 14 | 18 | 18 | 12 | 13 | 10 |  |
| Included area (%)^d^ | 84 | 50 | 81 | 100 | 83 | 87 | 54 |  |
| # individuals |  |  |  |  |  |  |  |  |
| Oct after 23^rd^ | 3 | 12 | 1 | 20 | 13 | 5 | 27 | 81 |
| Nov | 10 | 34 | 29 | 37 | 18 | 17 | 15 | 160 |
| Dec | 10 | 16 | 8 | 14 | 8 | 10 | 5 | 71 |
| Jan | 9 | 3 | 7 | 7 | 1 | 2 | 5 | 34 |
| Feb | 1 | 0 | 0 | 0 | 0 | 3 | 0 | 4 |
| Sample size A | **33** | **65** | **45** | **78** | **40** | **37** | **52** | **350** |
| Oct before 23^rd^ | 15 | 7 | 0 | 4 | 77 | 1 | 0 | 104 |
| Additional individuals ^e^ | 3 | 2 | 0 | 1 | 10 | 0 | 11 | 27 |
| Sample size B | **51** | **74** | **45** | **83** | **127** | **38** | **63** | **481** |

**Table S3.** Results from statistical test of whether the linear relationship between the ratio APR: (TNC2+lipids) and % fiber (Fig. 3) differed between the three diet types (DF = 313, adjusted R^2^ = 0.674).

|  | β | Std. error | t-value | p-value |
| --- | --- | --- | --- | --- |
| Intercept | 0.86 | 0.032 | 27.3 | < 0.001 |
| % fiber | -0.78 | 0.064 | -12.2 | < 0.001 |
| Broadleaf diet (B) | 0.13 | 0.042 | 3.1 | 0.002 |
| Conifer diet (C) | -0.09 | 0.081 | -1.1 | 0.285 |
| % fiber * B | -0.25 | 0.081 | -3.0 | 0.003 |
| % fiber * C | 0.13 | 0.145 | 0.9 | 0.382 |

**Table S4.** Principal component coefficients of the nutritional composition of 12 major food plant species for moose in Sweden. Included in the PCA were the concentrations (as % dm) of six nutritional constituents in the parts of the plants’ twigs that are edible for moose: available protein (Avail P: total N minus ADF-N), total non-structural carbohydrates (TNC1, by assay), in vitro digestible NDF (dNDF), cellulose, hemicellulose and lignin.

| Variable | PC1 | PC2 |
| --- | --- | --- |
| Lignin | 0,350 | 0,863 |
| Cellulose | 0,922 | -0,086 |
| Hemicellulose | 0,669 | -0,392 |
| Avail P | -0,151 | -0,946 |
| TNC1 | -0,952 | 0,099 |
| dNDF | 0,064 | -0,142 |
|  |  |  |
| % Variance | 39.2 | 30.5 |

**Table S5.** Principal component coefficients of the nutritional composition of five common supplementary feeds used in Sweden and 12 common moose food plants (parts of their twigs edible for moose). See PCA score plot in Fig. 5 Main text and loading plot in Fig. A3. Variables included are (as % dm) available protein (Avail P), neutral-detergent fiber (NDF) and total non-structural carbohydrates (TNC).

| Variable | PC1 | PC2 |
| --- | --- | --- |
| Avail P | 0,494 | 0,869 |
| TNC | 0,957 | -0,247 |
| NDF | -0,968 | 0,200 |
|  |  |  |
| % Variance | 69.9 | 28.6 |

**
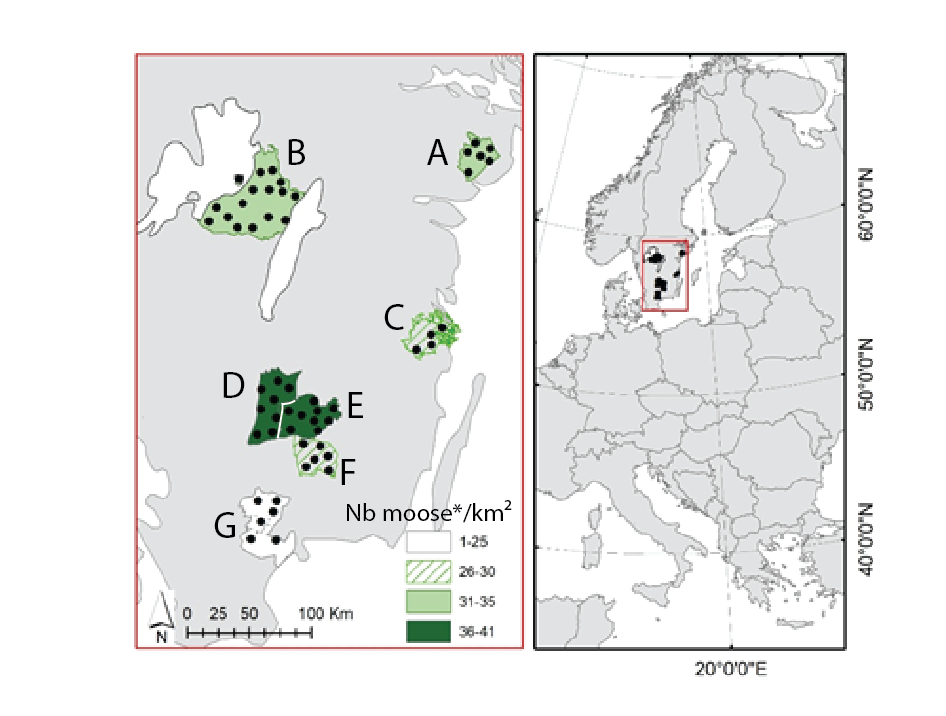
**

**Figure S1.** Map of the seven moose management areas (MMA) in Southern Sweden used as study areas. Each MMA harbours a moose population and includes moose management units (MMU, harbouring subpopulations) indicated with a black dot. Moose population density for each MMA during the study year is indicated with a coloured scale (*number of moose harvested in the yearly hunt km^-2^; sourced from [www.viltdata.se](http://www.viltdata.se)). Names of MMAs: A = Södermanland 3; B = Västra Götaland 6; C = Västervik Södra; D = Jönköping 6; E = Kronoberg 7; F = Kronoberg 4; G = Skåne Nordöstra.

**Figure S2.** Proportions of six different moose age-sex classes (where F = female, and M = male) in moose populations classified (by a previous study (Felton et al., 2020b)) as having one of three winter diet types. Age-sex proportions across diet types were not significantly different when assessed on the subpopulation level.

**
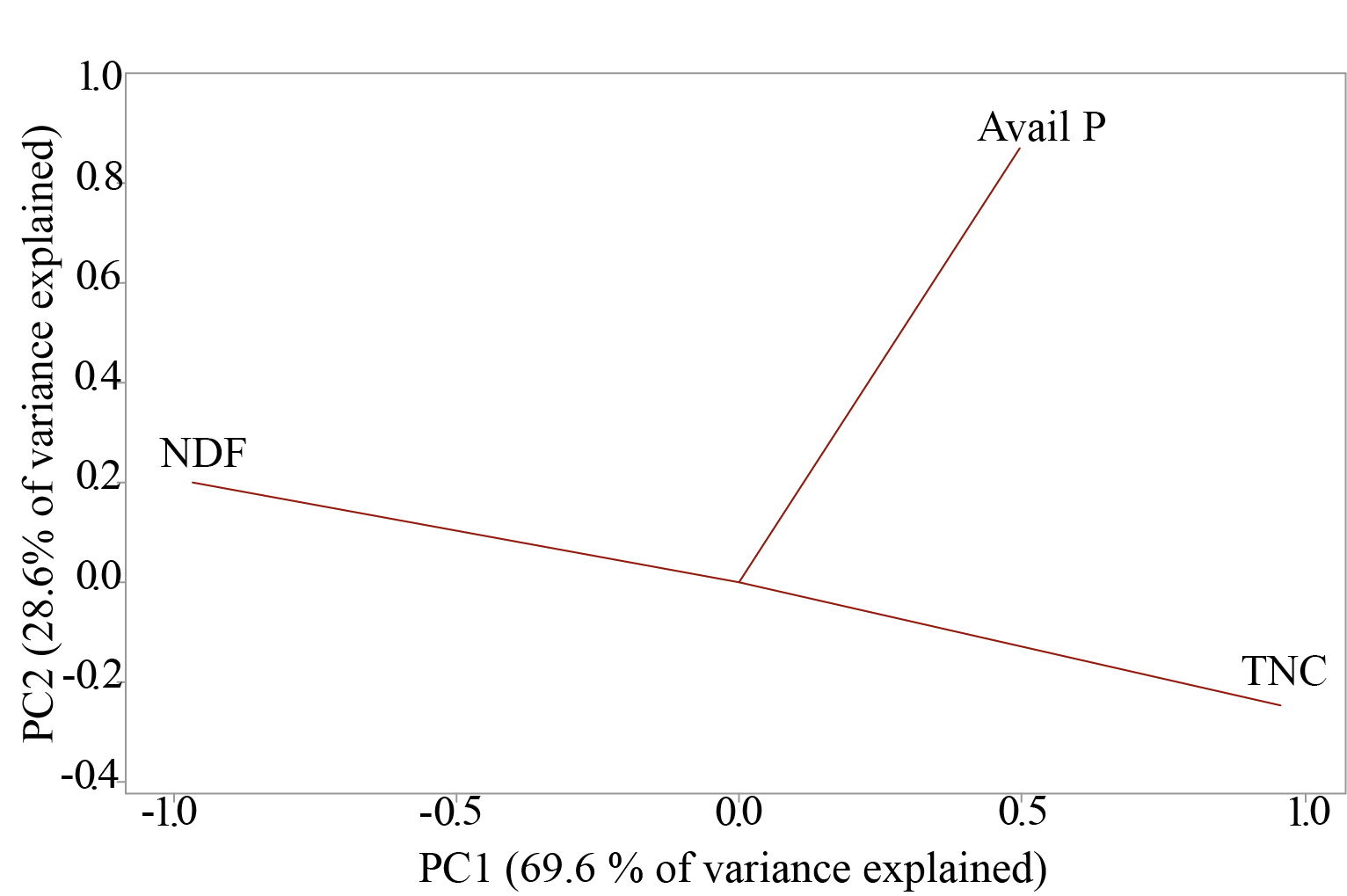
**

**Figure S3.** Loading plot from Principal Component Analysis (PCA) of the nutritional composition of five common supplementary feeds used in Sweden and 12 common moose food plants (parts of their twigs edible for moose). See PCA score plot in Fig. 5 Main text and principal component coefficients in Table A5. Included in this PCA are three nutritional constituents (as % dm): available protein (Avail P), NDF and total non-structural carbohydrates (TNC).
